# Hospital Out-Patient Reform and Provident Dispensaries

**Published:** 1890-01-25

**Authors:** Robert R. Rentoul

**Affiliations:** Liverpool


### Hospital Out-Patient Reform and Provident Dispensaries.

By Robert R. Rentoul, M.D., Liverpool.

In drawing up a table of payment for the benefit members, I would suggest that an "age table" be used as in other insurances. I would charge every member 6d. per month, and place the different rate of payment on the "entrance fee"; for, though the entrance fee would be paid yearly, each member would require to be examined by one of the medical staff before admission, and for this he would be paid 2s. out of the hon. members' fund.

The scale of yearly entrance fees would be—

| | s. | d. |
|---|---|---|
| From the age of 0 to 8 years | 2 | 0 |
| ,, ,, 8 to 16 ,, | 1 | 3 |
| ,, ,, 16 to 40 ,, | 0 | 6 |
| ,, ,, 40 to 55 ,, | 0 | 9 |
| ,, ,, 55 to 70 ,, | 1 | 0 |
| ,, ,, 80 and upwards | 2 | 6 |

When the entrance fee exceeds 1s. the balance to be paid from "hon. members' fund." One penny would be charged for each separate prescription. A levy of 1d. per month would be placed on each benefit member, so as to help in defraying management. The fees for vaccination, surgical or obstetric operations, and confinements would be the same as those of the public medical service. The sum subscribed by honorary members would be one-third the amount paid by benefit members. I would fine any doctor or dispenser 5s. who supplied any benefit member with treatment or medicines who was in arrear with payments. Members would enter into benefits two months after joining. In case of confined illness, or infirmity of old age, a yearly entrance fee of 5s. would be charged, plus the 6d. monthly and levy.

I think the above plan of placing the different scale of payments on an annual entrance fee is better than making different monthly payments for each member. It will be less cumbersome, and will induce members to stay. If the hospitals turn away at least 42·32 per cent. of their present out-patients the provident system would soon be a success. London, to begin with, would have a total of 700,000 benefit members in the first year.

Supposing we had a provident society of 1,000 benefit members the following would give a fair idea of the expenditure:

| | £ | s. | d. |
|---|---|---|---|
| Rent and taxes | 36 | 0 | 0 |
| Coal and gas | 3 | 0 | 0 |
| Printing, postage, and stationery | 12 | 0 | 0 |
| Drugs, bottles, bandages, instruments, etc. | 25 | 0 | 0 |
| Examination fees to medical staff | 100 | 0 | 0 |
| Cleaning of branch | 5 | 0 | 0 |
| Dispenser, including residence at branch | 65 | 0 | 0 |
| Commission on collection of members' payments, 5 per cent. | 15 | 0 | 0 |
| Part payment of entrance fees | 12 | 10 | 0 |
| Furnishing branch | 15 | 0 | 0 |
| Salaries to medical staff | 350 | 0 | 9 |
| | £637 | 10 | 0 |

| The income of such a branch would be: | £ | s. | d. |
|---|---|---|---|
| Sale of members' books, 2d. each | 8 | 6 | 8 |
| Fines | 1 | 0 | 0 |
| Sale of drugs | 20 | 6 | 8 |
| Levy on benefit members | 50 | 0 | 0 |
| Entrance fees at 1s. 3d. each (average) | 50 | 0 | 0 |
| Monthly payments from branch members | 300 | 0 | 0 |
| Subscriptions from honorary members | 214 | 16 | 8 |
| Total income | £644 | 10 | 0 |

thus leaving a balance of £7.

The above sample balance-sheet shows that with 1,000 members, we should require an income of £637, and to meet this, there would be a payment of £429 13s. 4d. from benefit members. A third of the latter sum must, therefore, come from honorary subscribers—that is, £214 16s. 8d.

As regards the amount, this would give to the medical staff of, say, four doctors, we should have from

| | £ | s. | d. |
|---|---|---|---|
| Examination fees from honorary subscribers | 100 | 0 | 0 |
| Part entrance fees | 12 | 10 | 0 |
| Entrance fees from benefit members | 50 | 0 | 0 |
| Monthly payments | 300 | 0 | 0 |
| | £462 | 10 | 0 |

This would give on an average £115 12s. 6d. to each doctor, and, giving five visits to each member, this would make a fee up to 1s. 10d.

The average yearly payment by each benefit member would be:

| | s. | d. |
|---|---|---|
| Average yearly entrance fee | 1 | 0 |
| Member's book | 0 | 2 |
| Twelve monthly subscriptions | 6 | 0 |
| Levy | 1 | 0 |
| Five prescriptions per annum | 0 | 5 |
| | 8 | 7 |

This is about 8d. per month, 2d. per week, or a farthing per diem; each benefit member would cost the society 12s. 10d. per annum, and would pay 8s. 7d. of this himself.

Some, especially employers of labour, will no doubt object to pay part, but such must be let know that medical men have gone as far as they can in supplying medical aid on a charitable basis. The limit of unpaid medical service has been reached. I do not think 1s. 10d. is too high a fee for a night visit. As at present conducted, the vast majority of provident dispensaries are supplying medical aid to very well-to-do people for a very few coppers a month. It is not too much to hope that as wages have gone up so much during the last few years, the industrial classes will see their way to cease asking for more medical charity. True charity begins at home, and, for my part, I cannot see why one deserving class of society should starve themselves, their wives, and children, and should leave them depending on one or two benevolent medical institutions so that well-to-do people may have more money to spend on luxuries. I am not now speaking of hospitals, where true charity should always reign, but of a class of the community who would be insulted if they were told they were, in a medical sense, paupers, and depending on the benevolent actions of others. I am speaking of a provident plan which should be as little depending on charity as are our Oddfellows' and Foresters' societies, or the German working classes under their compulsory plan of insurance against sickness.

